# Knowledge, Attitudes, and Antibiotic Prescribing Practices Among Physicians in Two High-Demand Healthcare Settings in Saudi Arabia

**DOI:** 10.3390/antibiotics15040376

**Published:** 2026-04-07

**Authors:** Nahla H. Hariri, Hanin Mohammed Alsaedi, Bayan Fawaz Alzahrani, Thekra Abdulhafith Alwafi, Khalid Abdulrahman Basamih, Donia Jamaan Alghamdi, Hadeel Abdullah Alolowi, Hanin Mahmoud Qadah, Maryam Abdulrahim Jadw, Safaa M. Alsanosi, Maram H. Alshareef, Mohammed A. Garout, Nizar S. Bawahab, Saleh A. K. Saleh, Heba M. Adly

**Affiliations:** 1 Department of Community Medicine and Pilgrims Healthcare, College of Medicine, Umm Al-Qura University, Makkah 21955, Saudi Arabia; mhashareef@uqu.edu.sa (M.H.A.); magarout@uqu.edu.sa (M.A.G.); 2 Department of Medicine and Surgery, College of Medicine, Taibah University, Madinah 42353, Saudi Arabia; haneen.moha.2003@gmail.com (H.M.A.); mariamjeddo@gmail.com (M.A.J.); 3 College of Medicine, Umm Al-Qura University, Makkah 21955, Saudi Arabia; bayanf.alzahrani@gmail.com (B.F.A.); thekraalwafi5@gmail.com (T.A.A.); khalidambn@gmail.com (K.A.B.); doniaalghamdi123@gmail.com (D.J.A.); 4 College of Medicine, Alrayan Medical College, Madinah 42214, Saudi Arabia; hadeelre09@gmail.com (H.A.A.); hanqadah@gmail.com (H.M.Q.); 5 Department of Pharmacology and Toxicology, College of Medicine, Umm Al-Qura University, Makkah 21955, Saudi Arabia; smsanosi@uqu.edu.sa; 6 Department of General Surgery, King Faisal Hospital, Ministry of Health, Makkah 24236, Saudi Arabia; nbawahab@moh.gov.sa; 7 Ministry of Health Branch in the Makkah Region, Makkah 24231, Saudi Arabia; 8 Directorate of Institutional Excellence, Batterjee Medical College, Jeddah 21442, Saudi Arabia; saleh.abdrabuh@bmc.edu.sa

**Keywords:** antimicrobial resistance, antibiotic stewardship, physician prescribing practices, knowledge, attitude, and practice (KAP), mass gathering healthcare

## Abstract

**Background:** Antimicrobial resistance continues to threaten effective infection management worldwide and is driven largely by inappropriate prescribing practices. In Saudi Arabia, the cities of Makkah and Al-Madinah experience intense seasonal healthcare demand due to the annual pilgrimage, creating additional challenges for rational antibiotic use. This study aimed to evaluate physicians’ knowledge, attitudes, and prescribing behaviors related to antibiotics in these high-demand settings. **Methods:** A cross-sectional analytic study was conducted between June and August 2024 among physicians practicing in Makkah and Al-Madinah, including those participating in Hajj services. A previously validated, structured electronic questionnaire assessed knowledge of common pathogens, perceptions of antimicrobial resistance, prescribing influences, and counseling practices. The survey was distributed electronically to eligible physicians. Descriptive statistics were generated, and associations were examined using appropriate inferential tests with a 95% confidence level. **Results:** A total of 487 physicians participated. Most respondents (74%) correctly identified major bacterial causes of upper respiratory tract infections, and 90% acknowledged the association between prior antibiotic exposure and resistance. Nonetheless, misconceptions persisted regarding the benefit of antibiotics in viral conditions. Workload and patient expectations influenced prescribing behavior; 77% reported a greater likelihood of prescribing antibiotics during periods of high clinical pressure. While adherence to guideline-based practice was generally reported, variability existed in counseling practices and perceptions of stewardship policies. **Conclusions:** Although baseline knowledge was satisfactory, contextual and behavioral factors continue to influence prescribing decisions and may contribute to unnecessary antibiotic exposure, highlighting the need for strengthened antimicrobial stewardship strategies in high-demand healthcare environments.

## 1. Introduction

Antimicrobial resistance (AMR) is recognized as one of the most serious global public health threats, driven largely by the misuse and overuse of antibiotics in both community and hospital settings [1]. Recent global estimates suggest that antimicrobial resistance contributes to nearly five million deaths annually, underscoring the urgent need to optimize antibiotic prescribing practices in both routine and high-demand healthcare environments [2]. Inappropriate antibiotic use contributes not only to resistance development but also to drug toxicity, adverse drug interactions, prolonged illness, therapeutic failure, and increased healthcare costs. Inappropriate antibiotic use contributes not only to resistance development but also to drug toxicity, adverse drug interactions, prolonged illness, therapeutic failure, and increased healthcare costs. Studies have shown that errors in antibiotic prescribing, whether related to indication, drug selection, dosage, administration interval, or duration, occur in approximately 30–50% of cases, even within hospital environments [2,3]. In intensive care units, unnecessary or suboptimal antibiotic use has been reported in up to 60% of prescriptions, further compounding the AMR burden [2].

To address these concerns, the World Health Organization (WHO) has emphasized strengthening prescriber awareness, improving antimicrobial stewardship practices, and implementing structured monitoring frameworks such as the AWaRe (Access, Watch, Reserve) classification system to guide rational antibiotic use [4]. Enhancing physicians’ knowledge and adherence to prescribing guidelines remains a central strategy in global AMR containment efforts [1,4].

Regions experiencing high healthcare demand present additional prescribing challenges. In Saudi Arabia, Makkah and Al-Madinah host millions of pilgrims annually during Hajj and Umrah seasons, resulting in substantial increases in patient volume and clinical workload. The Saudi Ministry of Health (MoH) has established health requirements and regulatory frameworks to ensure safe healthcare delivery during pilgrimage seasons [5]. However, the complexity of managing diverse infectious conditions in high-density settings underscores the importance of appropriate antibiotic prescribing practices.

Previous studies examining antibiotic knowledge and attitudes have demonstrated variable awareness levels among both healthcare providers and the public. Research conducted in different settings, including tertiary hospitals, medical schools, and community populations, has identified gaps in understanding antibiotic indications, resistance mechanisms, and appropriate use [2,6,7]. Among healthcare professionals, studies from Ethiopia and other regions have reported deficiencies in knowledge and beliefs regarding antimicrobial resistance [8].

Within Saudi Arabia, several investigations have evaluated physicians’ antibiotic prescribing practices. A study conducted among primary healthcare physicians in Jeddah revealed variability in knowledge, attitudes, and prescribing behaviors [9]. Similarly, research from the Qassim region demonstrated that physicians perceived antibiotic prescribing as a challenging and sometimes stressful clinical decision, with some participants questioning the necessity of laboratory confirmation before initiating therapy [10]. These findings indicate that, despite existing guidelines, gaps in knowledge and practice persist.

Recent global and regional reports (2024–2025) have further emphasized the need for continuous evaluation of prescribing behaviors, particularly in high-demand healthcare environments where diagnostic uncertainty, patient expectations, and time constraints may influence antibiotic decisions [11,12]. Strengthening antimicrobial stewardship in such contexts requires updated assessments of physicians’ knowledge, attitudes, and practices to inform targeted interventions.

Despite growing attention to antimicrobial stewardship in Saudi Arabia, contemporary data evaluating physicians’ awareness and prescribing behaviors in pilgrimage-hosting regions remain limited. Most previous studies have focused either on single institutions or on public perceptions rather than prescribing physicians operating under high clinical pressure.

Therefore, the primary objective of this study is to assess physicians’ knowledge, attitudes, and prescribing practices regarding antibiotic use and antimicrobial resistance in Makkah and Al-Madinah. Secondary objectives include evaluating knowledge related to bacterial infections and identifying factors influencing prescribing decisions. Understanding these dimensions is essential for guiding antimicrobial stewardship strategies in regions characterized by sustained healthcare demand.

## 2. Results

A total of 487 physicians participated in the study. The median age was 30 years (IQR 28–35). Most participants were male (66%) and Saudi nationals (90%). The majority were practicing in Al-Madinah (55%) or Makkah (40%), while 5.7% reported volunteering during the Hajj season. Regarding academic qualifications, 46% held a bachelor’s degree, 28% a master’s degree, 17% had a PhD/MD or completed fellowship training, and 8.8% held a diploma. Internal medicine was the most frequently reported specialty (20%), followed by general practice (16%), family medicine (14%), and pediatrics (13%). Nearly half of participants (45%) had less than five years of professional experience, whereas 15% had more than ten years (Table 1).

Concerning knowledge of common bacterial pathogens associated with respiratory tract infections, 74% identified *Streptococcus pneumoniae*, 66% identified *Haemophilus influenzae*, 49% selected *Moraxella catarrhalis*, and 33% selected *Staphylococcus aureus* Declines in infectious disease morbidity and mortality were most frequently attributed to vaccination (66%) and health awareness (63%), followed by antibiotic use (48%) and early diagnosis and treatment (43%). Most participants (85%) believed that antibiotics relieve symptoms, with 52% estimating the effect as great, 34% as moderate, and 14% as mild. For viral infections, 60% considered the preventive effect of antibiotics against secondary bacterial infection to be mild. The most commonly identified adverse consequences of antibiotic use were drug resistance (75%), disruption of normal flora (72%), and gastrointestinal disturbances (67%). The majority agreed that rational prescribing reduces side effects (88%), whereas 91% stated that irrational use increases adverse effects. Overuse (76%) and misuse (72%) were perceived as the principal contributors to antimicrobial resistance (Table 2).

Attitudinal responses indicated that 90% believed prior antibiotic use increases the risk of drug-resistant infection. More than half (53%) reported feeling pressured when patients expected antibiotic prescriptions, and 77% acknowledged being more likely to prescribe antibiotics during periods of heavy workload. Approximately 64% agreed that patients may perceive their illness as not being taken seriously if antibiotics are not prescribed. Institutional antibiotic guidelines were reported as available by 88% of respondents, and 94% considered guideline use essential for appropriate patient care. However, 54% believed that existing guidelines do not sufficiently account for individual patient variation. Sixty-four percent supported restriction of antibiotic prescriptions without laboratory confirmation of bacterial infection (Table 3).

With regard to prescribing practices, 72% stated that they always advise patients to complete the full course of antibiotics, and 58% always inform patients that proper antibiotic use shortens illness duration. Only 11% consistently advised stopping antibiotics once symptoms improve, while 67% reported always advising against sharing leftover antibiotics. Side effects were routinely discussed by 63% of participants (Table 4).

Most physicians (64%) recommended taking antibiotics after meals. Regarding storage, 38% tailored advice according to antibiotic type, 33% recommended room temperature storage, and 22% advised refrigeration. In cases of adverse effects, 82% advised immediate medical consultation. Inquiry about pregnancy status was reported as “always” by 53% of respondents, and 72% consistently asked about allergy history. When a dose was missed, 69% advised resuming the next scheduled dose, whereas 19% recommended taking it when remembered and 13% advised doubling the next dose (Table 5).

Age was significantly associated with perceived pressure to prescribe antibiotics (*p* < 0.001) and the belief that patients would feel dismissed without an antibiotic prescription (*p* = 0.008). Other age-related comparisons were not statistically significant (Table 6).

Gender differences were observed in perceptions regarding patient satisfaction if antibiotics were not prescribed (*p* = 0.007), the anticipated impact of patient education (*p* = 0.034), and the perceived limitations of guidelines in addressing individual patient variation (*p* = 0.004). No significant gender associations were found for other variables (Table 7).

Professional experience was associated with perceptions regarding side effects. Physicians with more than ten years of experience were more likely to believe that adherence minimizes adverse effects (92%; *p* = 0.016) and that irrational antibiotic use increases side effects (99%; *p* = 0.010). Other associations with years of experience were not statistically significant (Table 8).

A multivariable logistic regression model was constructed to assess factors independently associated with feeling pressured to prescribe antibiotics. After adjustment, age demonstrated a borderline association with perceived prescribing pressure (adjusted OR = 1.03 per year increase, 95% CI: 1.00–1.05; *p* = 0.053). Gender was not significantly associated with perceived pressure (adjusted OR = 1.05; 95% CI: 0.72–1.53; *p* = 0.795) (Table 9).

**Figure 1:**
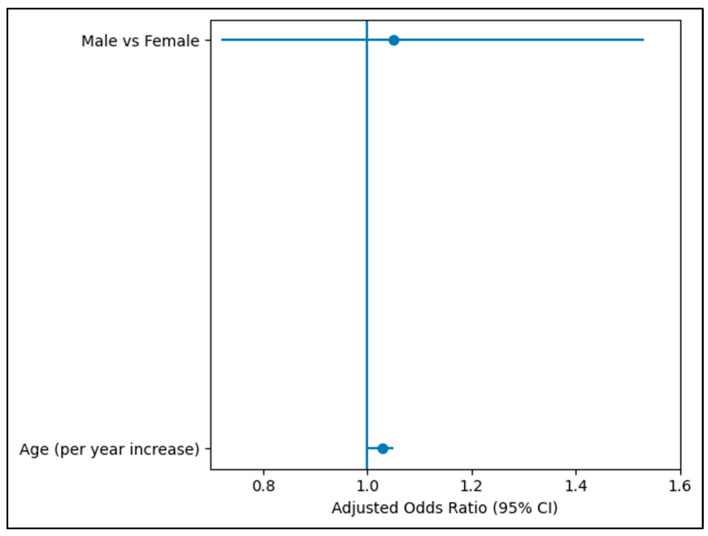
Adjusted Odds Ratios for Factors Associated with Feeling Pressured to Prescribe Antibiotics.

Figure 1 Forest plot of adjusted odds ratios (AORs) for factors associated with physicians feeling pressured to prescribe antibiotics. The vertical line represents an odds ratio (OR) of 1.0 (no association). Age was not significantly associated with perceived prescribing pressure (adjusted OR = 1.03 per year increase, 95% CI: 1.00–1.05; *p* = 0.053). Male physicians did not differ significantly from female physicians (AOR = 1.05; 95% CI: 0.72–1.53; *p* = 0.795). Confidence intervals crossing 1.0 indicate a lack of statistical significance. Although age demonstrated did not reach statistical significance in the unadjusted analysis, it did not reach conventional statistical significance after adjustment.

## 3. Discussion

This study assessed physicians’ knowledge, attitudes, and antibiotic prescribing practices in two high-demand healthcare environments in Saudi Arabia. Overall, physicians demonstrated satisfactory foundational knowledge regarding antimicrobial resistance and common bacterial pathogens; however, prescribing decisions were frequently influenced by contextual factors such as workload pressure and patient expectations. High-intensity healthcare environments such as the Hajj pilgrimage present unique antimicrobial stewardship challenges due to sudden surges in patient volume, diagnostic uncertainty, and time pressure on clinicians. These contextual pressures may lower prescribing thresholds and increase the likelihood of unnecessary antibiotic use, highlighting the need for structured stewardship interventions in such settings.

### 3.1. Clinical Knowledge and Foundational Competence

Antibiotics remain indispensable in the management of bacterial infections; however, their clinical value depends entirely on appropriate selection, dosing, duration, and patient-specific considerations. The present study demonstrates that most participating physicians correctly identified common bacterial pathogens responsible for respiratory tract infections, including *Streptococcus pneumoniae*, *Haemophilus influenzae*, and *Moraxella catarrhalis*. This indicates adequate foundational microbiological knowledge. Nevertheless, the epidemiology of URTIs is dynamic and frequently viral in origin, and inappropriate empirical antibiotic prescribing remains common globally.

The safe use of antibiotics in special populations particularly pregnant women remains a critical area of practice. Antibiotic exposure during pregnancy must be carefully evaluated because certain agents, such as tetracyclines and fluoroquinolones, are associated with fetal risks, whereas beta-lactams and selected other agents are generally considered safer alternatives when clinically indicated [13]. Routine inquiry about pregnancy status before prescribing antibiotics is therefore not only a clinical best practice but also a patient safety imperative. Our findings suggest variability in this regard, indicating that safety screening protocols should be strengthened within prescribing workflows.

### 3.2. Perceived Benefits, Misconceptions, and Secondary Bacterial Infections

Although the majority of physicians acknowledged the role of vaccination and health awareness in reducing infectious disease burden, a proportion still perceived antibiotics as beneficial for preventing secondary bacterial infections. This reflects a nuanced misconception. Prevention of secondary bacterial infection is not achieved through prophylactic antibiotic use in uncomplicated viral illness; rather, it depends on appropriate management and monitoring of the primary infection. Unwarranted prophylactic prescribing may paradoxically increase resistance selection pressure. These findings highlight the need to reinforce updated evidence-based guidance regarding indications for antibiotic therapy.

### 3.3. Communication, Gender Differences, and Patient-Centered Care

Gender-associated differences were observed in perceptions related to patient expectations and education. Evidence from primary care settings indicates that communication style and empathic engagement may vary by physician gender, potentially influencing patient interactions and decision-making processes [14,15,16]. Female physicians often demonstrate higher empathic communication patterns, whereas male physicians may emphasize directive clinical decision-making. These patterns may influence how physicians interpret and respond to perceived patient pressure [17].

However, stewardship effectiveness depends less on demographic characteristics and more on structured communication strategies. Training physicians to manage expectations through clear explanations, delayed prescribing approaches when appropriate, and safety-netting advice has been shown to reduce unnecessary antibiotic prescriptions without compromising patient satisfaction [18,19]. Thus, communication skills training should be integrated into antimicrobial stewardship programs (ASPs).

### 3.4. Workload, Patient Expectations, and Prescribing Pressure

One of the most notable findings in this study is the influence of workload and perceived patient expectations on prescribing behavior. A substantial proportion of physicians reported being more likely to prescribe antibiotics under high workload conditions. This aligns with international evidence demonstrating that contextual pressures including time constraints, consultation fatigue, and perceived demand lower prescribing thresholds even when clinical indication is uncertain [20].

Importantly, stewardship research increasingly emphasizes that inappropriate prescribing is rarely driven by knowledge deficits alone; rather, it emerges from system-level and behavioral factors. Experienced antimicrobial stewardship leaders note that prescribing behavior must be addressed through structural interventions rather than relying solely on educational messaging [21]. Therefore, addressing workload-associated prescribing pressure requires institutional support, workflow redesign, and real-time decision support mechanisms.

An important observation in this study is that many physicians indicated that a patient’s financial situation can influence the choice of antibiotics. This finding likely reflects the realities of everyday clinical practice, where the cost and availability of medications, as well as the patient’s ability to afford them, can affect adherence to treatment. In such situations, physicians may opt for more affordable or readily available options, even if other antibiotics might be more appropriate from a clinical perspective. While this approach may support patient adherence, it can also lead to variation in prescribing practices and highlights the importance of ensuring equitable access to effective antimicrobial therapy.

### 3.5. Years of Experience and Knowledge Gradients

The study demonstrated that physicians with more than ten years of experience showed stronger agreement regarding the relationship between irrational antibiotic use and adverse outcomes. This is consistent with evidence suggesting that cumulative clinical exposure enhances risk recognition and prescribing confidence. However, the observed knowledge variability among mid-career physicians suggests the need for targeted continuing professional development interventions.

Modern medical education literature supports competency-based and case-driven training approaches rather than passive educational sessions [22]. Continuous professional development that incorporates local resistance patterns, case audits, and feedback can bridge these gaps and standardize prescribing behaviors across experience levels.

### 3.6. Practical Counseling: Storage, Missed Doses, and Adherence

Practical aspects of antibiotic counseling were variably addressed by participants. Proper storage of reconstituted oral antibiotic suspensions is essential to maintain pharmacological stability and therapeutic efficacy. Certain formulations require refrigeration to preserve potency, while others remain stable at room temperature; inappropriate storage may lead to reduced efficacy and perceived treatment failure [14].

Similarly, guidance regarding missed doses and course completion remains central to adherence and resistance prevention. Although most participants emphasized completion of therapy, modern stewardship also supports evidence-based shorter treatment durations where appropriate. Rational prescribing therefore requires balancing adequate duration with minimizing unnecessary exposure.

### 3.7. From Knowledge to Systems: The Stewardship Imperative

While awareness levels were generally satisfactory, awareness alone does not translate into optimal prescribing behavior. Antimicrobial stewardship programs are designed to operationalize rational antibiotic use through structured accountability, guideline implementation, audit and feedback, and multidisciplinary oversight [16,23].

1. Recent stewardship models emphasize several core components;
2. Leadership commitment and accountability;
3. Evidence-based prescribing pathways aligned with resistance data;
4. Diagnostic stewardship to reduce uncertainty-driven prescribing;
5. Prospective audit and feedback;
6. Education is integrated with real-time clinical decision support;
7. Measurement of prescribing indicators.

Global frameworks such as the World Health Organization (WHO) AWaRe classification provide structured guidance for antibiotic selection by promoting the preferential use of “Access” antibiotics and restricting unnecessary prescribing of “Watch” and “Reserve” agents to reduce resistance pressure while preserving clinical effectiveness [24,25]. The AWaRe framework was developed as part of the WHO Global Action Plan on Antimicrobial Resistance to support stewardship implementation and national monitoring of antibiotic consumption patterns [24]. Recent analyses have demonstrated that increasing the proportion of Access-group antibiotics within hospital formularies is associated with improved stewardship performance indicators and lower resistance selection pressure [25]. Incorporating AWaRe principles into institutional prescribing pathways, particularly in high-demand settings, may therefore strengthen antimicrobial stewardship efforts while maintaining therapeutic efficacy. Thus, incorporating the WHO AWaRe classification into institutional prescribing pathways may support more rational antibiotic selection and reduce resistance selection pressure.

### 3.8. Proposed Framework for Rational Antibiotic Use

**Figure 2:**
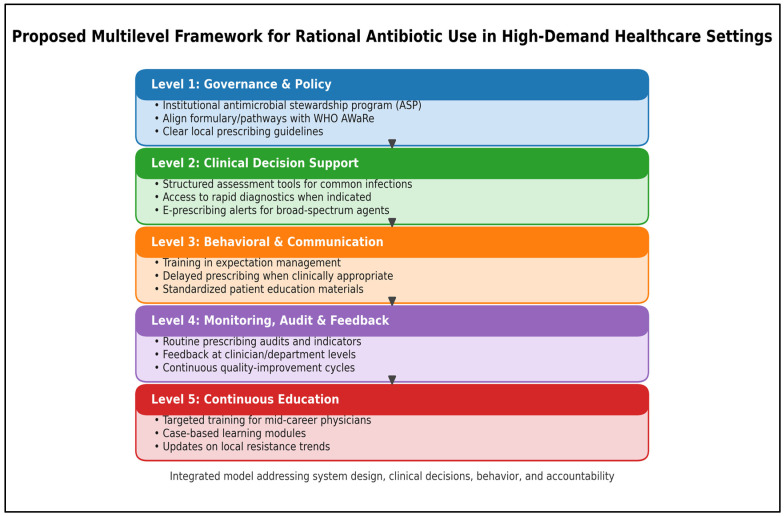
Proposed Multilevel Framework for Rational Antibiotic Use in High-Demand Healthcare Settings (Makkah and Al-Madinah).

Based on the findings of this study and contemporary stewardship principles, in Figure 2, we propose this framework synthesizes the study findings with contemporary antimicrobial stewardship principles by organizing interventions into five linked levels: (1) Governance & Policy (formal ASP adoption, local guidelines, and alignment with the WHO AWaRe classification), (2) Clinical Decision Support (structured assessment tools, access to diagnostics, and electronic alerts for broad-spectrum prescribing), (3) Behavioral & Communication (training in expectation management, delayed prescribing when appropriate, and standardized education materials), (4) Monitoring, Audit & Feedback (routine audits, prescriber/department feedback, and quality-improvement cycles), and (5) Continuous Education (targeted upskilling especially for mid-career physicians and updates on local resistance trends). The model emphasizes that sustainable improvement requires coordinated system-level design and accountability rather than relying only on individual knowledge [26,27,28].

## 4. Subjects and Methods

### 4.1. Study Design and Setting

A cross-sectional analytic study was conducted between June and August 2024 in Makkah and Al-Madinah, Saudi Arabia. Makkah and Al-Madinah are the two holiest cities in Saudi Arabia and host some of the largest recurring mass gatherings worldwide. In recent years, Hajj has brought approximately 1.8–2.5 million pilgrims annually within a few days, while Umrah attracts more than 10 million visitors each year across all seasons. Under Saudi Vision 2030, the Kingdom aims to increase the number of Umrah pilgrims to 30 million annually by 2030. These sustained and seasonal population surges place exceptional pressure on healthcare systems, generating a high-intensity clinical environment that demands efficient, evidence-based medical practice, including rational and careful antibiotic prescribing.

### 4.2. Study Population

The study targeted licensed physicians aged ≥24 years who were practicing in hospitals located in Makkah or Al-Madinah, including those participating in healthcare services during the Hajj season.

### 4.3. Inclusion and Exclusion Criteria

Eligible participants were physicians who routinely prescribe antibiotics as part of their clinical duties. These included physicians working in hospital settings in Makkah or Al-Madinah and those involved in Hajj-related healthcare services.

Medical students, interns, dentists, and healthcare providers not authorized to prescribe antibiotics were excluded. Physicians whose specialties do not typically involve antibiotic prescribing (e.g., radiology and psychiatry) were also excluded.

### 4.4. Sample Size Calculation

The minimum required sample size was calculated using the RaoSoft^®^ sample size calculator [29]. Based on an estimated target population of 8887 physicians, as reported in the Ministry of Health Statistical Annual Yearbook 2022 [30], the following assumptions were applied: 95% confidence level, anticipated response distribution of 50%, and design effect of 1. The calculated minimum sample size was 369 participants. To account for potential incomplete responses, the target sample was increased to 400 participants.

### 4.5. Study Instrument

Data were collected using a structured, self-administered electronic questionnaire in English. The instrument was adapted from a previously validated survey assessing physicians’ knowledge of antibiotic prescribing in respiratory tract infections in primary healthcare centers in Qassim [10]. The questionnaire was reviewed for clarity and contextual relevance before distribution. It comprised three sections: informed consent, demographic information, and items assessing knowledge, attitudes, and practices related to antibiotic use.

### 4.6. Study Procedures

The questionnaire was developed using Google Forms and distributed electronically. Eligible physicians received the survey link along with a brief explanation of the study objectives. Participation was voluntary. The survey link was circulated through professional communication channels and social media platforms commonly used by physicians in the target cities. Data collection was monitored to ensure completeness and data integrity.

### 4.7. Data Collection and Statistical Analysis

Data was analyzed using the Statistical Package for the Social Sciences (SPSS), version 22.0 (IBM Corp., Armonk, NY, USA). Continuous variables were presented as mean ± standard deviation (SD) for normally distributed data or median with range for non-normally distributed data. Categorical variables were summarized as frequencies and percentages. Some questionnaire items allowed multiple responses. Therefore, percentages for these variables may exceed 100%. Missing responses were excluded from analysis on a per-item basis.

Comparisons between groups were performed using the independent Student’s *t*-test or Mann–Whitney U test for continuous variables, as appropriate. Associations between categorical variables were assessed using the Chi-square test. A two-tailed *p*-value < 0.05 was considered statistically significant. The confidence interval (CI) was set at 95%.

### 4.8. Ethical Considerations

Ethical approval was obtained from the Institutional Review Board of Umm Al-Qura University (Approval No. HAPO-02-K-012-2024-10-2249) prior to study initiation. Participation was voluntary, and responses were collected anonymously. No identifying information was obtained. Data were stored securely and accessible only to the research team. Confidentiality was maintained throughout all stages of the study.

## 5. Limitations and Future Directions

This study has limitations that should be acknowledged. The reliance on self-administered responses introduces the possibility of reporting bias, as participants may unintentionally overstate adherence to recommended prescribing practices or their level of knowledge. The cross-sectional nature of the design limits interpretation to associations observed at a single time point and does not permit conclusions regarding causal pathways or changes over time. Furthermore, the sample was confined to physicians practicing in Makkah and Al-Madinah. Although these cities represent high-demand healthcare settings, regional variation across the Kingdom may limit the broader applicability of the findings. Additionally, some questionnaire items did not include all clinically appropriate response options. For example, the question regarding antibiotic intake in relation to meals did not include the option “depends on the antibiotic,” although this is clinically relevant. This may have influenced respondents’ answers and limited the interpretation of this finding. Notwithstanding these limitations, the results highlight specific domains that require attention. Strengthening formal training in antimicrobial prescribing at both undergraduate and postgraduate levels may address identified knowledge gaps. Periodic Continuing Medical Education activities grounded in current national and international recommendations, together with updates on local resistance trends, would further support appropriate prescribing behavior. Institutional antimicrobial stewardship programs, supported by clearly defined local protocols, remain central to optimizing antibiotic use.

Routine monitoring of prescribing patterns, coupled with structured feedback mechanisms, may enhance compliance with established guidelines. Educational efforts targeting both clinicians and patients should aim to clarify appropriate indications for antibiotic therapy and address misconceptions regarding their effectiveness. When clinically feasible, encouraging microbiological confirmation before initiation of broad-spectrum agents may contribute to more precise treatment decisions.

Future investigations should evaluate system-level strategies designed to reduce pressure in high-workload environments. The integration of clinical decisions supports tools and audit-driven feedback systems warrants further exploration. Additionally, research focusing on communication strategies that align patient expectations with evidence-based care may help mitigate demand-driven antibiotic use.

## 6. Conclusions

This study provides an updated assessment of physicians’ knowledge, attitudes, and prescribing practices regarding antibiotic use and antimicrobial resistance in two of the Kingdom’s highest-demand healthcare settings. Overall, physicians demonstrated satisfactory foundational knowledge of common bacterial pathogens and acknowledged the risks associated with irrational antibiotic use. However, prescribing behavior was influenced by contextual factors, particularly workload pressure and perceived patient expectations.

Although awareness of antimicrobial resistance was high, gaps were observed in areas related to practical counseling, individualized application of guidelines, and management of patient-driven demand. Age and professional experience showed limited influence on perceived prescribing pressure after multivariable adjustment, indicating that structural and system-level determinants may play a more decisive role than individual characteristics.

The findings underscore that improving antibiotic use requires more than knowledge reinforcement. Sustainable progress depends on strengthening institutional antimicrobial stewardship programs, integrating decision-support tools into routine practice, aligning prescribing pathways with WHO AWaRe principles, and implementing continuous audit and feedback mechanisms.

In high-demand environments such as Makkah and Al-Madinah, where clinical workload is substantial and patient turnover is high, structured stewardship interventions are essential to safeguard therapeutic effectiveness while limiting resistance selection pressure. A coordinated, multilevel framework that integrates governance, clinical decision support, behavioral strategies, monitoring systems, and continuous education may provide a practical pathway toward rational antibiotic use and long-term antimicrobial resistance containment.

## Figures and Tables

**Table 1:** Demographic and professional characteristics (*n* = 487).

Characteristic	*n* (%)
Age (Years)	30 (28, 35)
Sex	
Female	167 (34%)
Male	320 (66%)
Nationality	
Non-Saudi	48 (9.8%)
Saudi	439 (90%)
Place of work	
Al-Madinah	266 (55%)
Makkah	193 (40%)
Volunteering in Hajj	28 (5.7%)
Degree	
Bachelor’s degree	223 (46%)
Diploma	43 (8.8%)
Master’s degree	136 (28%)
PhD/MD/Fellowship	85 (17%)
Specialty	
Internal medicine	99 (20%)
General practitioner	77 (16%)
Family medicine	68 (14%)
Pediatric	66 (13%)
Emergency medicine	60 (12%)
General surgery	49 (9.9%)
* Obs/GYN	24 (4.8%)
* ENT	21 (4.2%)
Others	19 (4.0%)
Orthopedics	4 (0.8%)
Experience	
Less than 5 years	219 (45%)
5–10 years	193 (40%)
More than 10 years	75 (15%)

* Obs/GYN: Obstetrics and Gynecology; ENT: Ear, Nose, and Throat.

**Table 2:** Knowledge of antibiotics among physicians (*n* = 487).

Characteristic	*n* (%)
Common bacterial pathogens causing respiratory tract infections *	
*Streptococcus pneumoniae*	398 (81.7%)
*Haemophilus influenzae*	322 (66.1%)
*Moraxella catarrhalis*	239 (49.1%)
*Staphylococcus aureus*	162 (33.3%)
Atypical bacteria	110 (22.6%)
Group A beta-hemolytic streptococci	92 (18.9%)
Viridans streptococci	36 (7.4%)
Factors contributing to the decline in morbidity and mortality from infectious diseases *	
Vaccination	322 (66.1%)
Health awareness and education	305 (62.6%)
Use of antibiotics	234 (48.0%)
Early diagnosis and treatment	212 (43.5%)
Improvement of nutrition and hygiene	184 (37.8%)
Technological improvement	111 (22.8%)
Antibiotics lead to relief of symptoms	
No	72 (14.8%)
Yes	415 (85.2%)
Estimated effect of antibiotics on symptom relief	
Great	253 (52.0%)
Moderate	165 (33.9%)
Mild	69 (14.2%)
Effect of antibiotics in preventing secondary bacterial infection in viral illness	
Great	48 (9.9%)
Moderate	143 (29.4%)
Mild	296 (60.8%)
Common side effects of antibiotic use *	
Drug resistance	363 (74.5%)
Disruption of normal flora (reduced immunity)	350 (71.9%)
Gastrointestinal upset	326 (66.9%)
Allergic reactions	227 (46.6%)
General symptoms (e.g., headache, fever)	48 (9.9%)
Taking antibiotics as prescribed minimizes side effects	
Yes	429 (88.1%)
No	58 (11.9%)
Taking antibiotics without rational indication increases side effects	
Yes	443 (91.0%)
No	44 (9.0%)
Factors contributing to antibiotic resistance *	
Overuse of antibiotics	368 (75.6%)
Misuse of antibiotics	353 (72.5%)
Patient noncompliance	181 (37.2%)
Inadequate sanitation and hygiene	79 (16.2%)

* Percentages are calculated based on available responses. Some questions allowed multiple answers; therefore, totals may exceed 100%. Severity categories were self-reported by participants and reflect perceived clinical importance.

**Table 3:** Attitude towards antibiotic use among physicians.

Characteristic	No	Yes
Do you think that previous use of antibiotics increases a person’s risk of acquiring drug resistance infection?	51 (10%)	436 (90%)
Do you feel under pressure if your patient expects an antibiotic prescription	227 (47%)	260 (53%)
If your workload is high, are you more likely to prescribe antibiotics to relieve patient worry quickly	375 (77%)	112 (23%)
Do you think that if a prescription is issued, the consultation will be short	251 (52%)	236 (48%)
If you do not prescribe antibiotics, patients will feel their illnesses are not taken seriously.	176 (36%)	311 (64%)
Do you think that educating patient regarding antibiotic use will affect their expectation in later consultation	68 (14%)	419 (86%)
The patient’s economic condition affects your selection of the prescribed antibiotics	123 (25%)	364 (75%)
Are there guidelines regarding antibiotic use in your institution	59 (12%)	428 (88%)
If yes, do these guidelines affect your selection of the prescribed antibiotics	46 (11%)	378 (89%)
I believe using guidelines for antibiotics use is needed for proper patient care.	30 (6.2%)	457 (93.8%)
The available guidelines for antibiotic use do not consider the individual variation of patients’ needs.	224 (46%)	263 (54%)
Do you support a regulation prohibiting antibiotic prescription without laboratory confirmation of bacterial infection?	177 (36%)	310 (64%)

Percentages are calculated based on available responses; totals may not equal the overall sample size due to missing data.

**Table 4:** Practices of antibiotic prescription among physicians.

Characteristic	Always	Usually	Sometimes	Never
Which of the following advice do you give to him/her?				
Proper use of antibiotics shortens the duration of illness	282 (58%)	156 (32%)	42 (8.6%)	7 (1.4%)
When you feel better, stop taking the further antibiotic	55 (11%)	110 (23%)	64 (13%)	258 (53%)
Save the remaining antibiotics for the next time you get sick	47 (9.7%)	67 (14%)	66 (14%)	307 (63%)
Discard the remaining leftover antibiotic	151 (31%)	132 (27%)	146 (30%)	58 (12%)
You can give the leftover antibiotic to your friend/family member if they get sick	42 (8.6%)	56 (11%)	64 (13%)	325 (67%)
Complete the full course of treatment	349 (72%)	91 (19%)	41 (8.4%)	6 (1.2%)
Do not repeat the treatment without consultation	298 (61%)	123 (25%)	58 (12%)	8 (1.6%)
Do not stop the treatment without consultation	298 (61%)	135 (28%)	47 (9.7%)	7 (1.4%)
Do not decrease or increase the dosage without consultation	323 (66%)	110 (23%)	48 (9.9%)	6 (1.2%)
Inform him/her about the side effects of the prescribed antibiotic	306 (63%)	121 (25%)	54 (11%)	6 (1.2%)

**Table 5:** Advice related to antibiotic prescription (*n* = 487).

Characteristic	*n* = 487
Advice to the patient to take the prescribed antibiotic in relation to meal	
After meal	310 (64%)
Before meal	86 (18%)
During meal	53 (11%)
It does not matter	38 (7.8%)
Advice to the patient regarding the storage of the prescribed antibiotic	
According to the type	187 (38%)
Cold, dry place away from pediatric	1 (0.2%)
In a place exposed to the sun	32 (6.6%)
In the refrigerator	108 (22%)
In the room temperature	159 (33%)
Advice when patient experienced adverse effects	
Consult the doctor immediately	397 (82%)
Stop taking the antibiotic	256 (53%)
Induce vomiting	70 (14%)
Ask women about her pregnancy status when prescribing antibiotic	
Always	258 (53%)
Never	24 (5.0%)
Occasionally	93 (19%)
Only if antibiotics can affect fetus	112 (23%)
Ask your patient about history of allergies when prescribe antibiotic	
Always	353 (72%)
Never	27 (5.5%)
Occasionally	69 (14%)
Only if antibiotics can cause allergy	38 (7.8%)
Advice to patients who miss a dose	
Double the next dose	62 (13%)
Resume with the next dose	334 (69%)
Take the dose when you remember	91 (19%)

Percentages are calculated based on available responses. Some questions allowed multiple answers; therefore, totals may exceed 100%.

**Table 6:** Association between age (median, IQR) and knowledge and attitudes toward antibiotic use.

Characteristic	*n* ^1^ = 487	*p*-Value ^2^
If antibiotics are taken as prescribed will this minimize the side effects		0.3
No	31 (28, 36)	
Yes	30 (28, 35)	
If antibiotics are taken without rational indication, will this increase the side effects		0.2
No	30 (28, 32)	
Yes	30 (28, 35)	
Do you think that previous use of antibiotic increases person risk of acquiring drug resistance infection		0.2
No	30 (29, 34)	
Yes	30 (28, 35)	
Feel under pressure if your patient expects antibiotic prescription		<0.001
No	29 (28, 32)	
Yes	31 (28, 36)	
If your work load is high, are you more likely to prescribe antibiotics to relieve patient worry quickly		0.089
No	30 (28, 35)	
Yes	30 (28, 32)	
Think that if a prescription is issued, the consultation will be short		0.8
No	30 (28, 35)	
Yes	30 (28, 34)	
If you do not prescribe antibiotics, patients will feel their illnesses are not taken seriously		0.008
No	29 (28, 33)	
Yes	31 (28, 36)	
Think that educating patient regarding antibiotic use will have an effect on their expectation in later consultation		0.8
No	30 (28, 32)	
Yes	30 (28, 35)	
Patient economic condition affects your selection of the prescribed antibiotics		0.5
No	31 (28, 36)	
Yes	30 (28, 34)	
Is there guidelines regarding antibiotic use in your institution		0.2
No	30 (28, 34)	
Yes	30 (28, 35)	
If yes, do these guidelines affect your selection of the prescribed antibiotics		0.5
No	30 (28, 33)	
Yes	30 (28, 35)	
Unknown	63	
In my opinion, use of guidelines for antibiotics use are needed for proper patient care		0.9
No	30 (28, 36)	
Yes	30 (28, 35)	
The available guidelines for antibiotics use do not consider the individual variation of patients needs		0.7
No	30 (28, 35)	
Yes	30 (28, 35)	
Support a regulation to prohibit antibiotic prescription without laboratory confirmation of bacterial infection		0.2
No	31 (28, 36)	
Yes	30 (28, 34)	

^1^ Age: Median (IQR); ^2^ Wilcoxon rank sum test; Kruskal–Wallis rank sum test. Age is presented as median (IQR). Comparisons were performed using the Wilcoxon rank-sum test or Kruskal–Wallis test as appropriate.

**Table 7:** Association between gender, knowledge and attitude towards antibiotics.

Characteristic	Female, *n* ^1^ = 167	Male, *n* = 320	*p*-Value ^2^
If antibiotics are taken as prescribed will this minimize the side effects			0.6
No	18 (11%)	40 (13%)	
Yes	149 (89%)	280 (88%)	
If antibiotics are taken without rational indication, will this increase the side effects			0.3
No	18 (11%)	26 (8.1%)	
Yes	149 (89%)	294 (92%)	
Do you think that previous use of antibiotic increases person risk of acquiring drug resistance infection			0.2
No	22 (13%)	29 (9.1%)	
Yes	145 (87%)	291 (91%)	
Feel under pressure if your patient expects antibiotic prescription			0.4
No	82 (49%)	145 (45%)	
Yes	85 (51%)	175 (55%)	
If your work load is high, are you more likely to prescribe antibiotics to relieve patient worry quickly			0.15
No	135 (81%)	240 (75%)	
Yes	32 (19%)	80 (25%)	
Think that if a prescription is issued, the consultation will be short			0.3
No	81 (49%)	170 (53%)	
Yes	86 (51%)	150 (47%)	
If you do not prescribe antibiotics, patients will feel their illnesses are not taken seriously			0.007
No	74 (44%)	102 (32%)	
Yes	93 (56%)	218 (68%)	
Think that educating patient regarding antibiotic use will have an effect on their expectation in later consultation			0.034
No	31 (19%)	37 (12%)	
Yes	136 (81%)	283 (88%)	
Patient economic condition affects your selection of the prescribed antibiotics			0.3
No	47 (28%)	76 (24%)	
Yes	120 (72%)	244 (76%)	
Is there guidelines regarding antibiotic use in your institution			0.092
No	26 (16%)	33 (10%)	
Yes	141 (84%)	287 (90%)	
If yes, do these guidelines affect your selection of the prescribed antibiotics			0.4
No	13 (9.2%)	33 (12%)	
Yes	128 (91%)	250 (88%)	
Unknown	26	37	
In my opinion, use of guidelines for antibiotics use are needed for proper patient care			0.3
No	13 (7.8%)	17 (5.3%)	
Yes	154 (92%)	303 (95%)	
The available guidelines for antibiotics use do not consider the individual variation of patients needs			0.004
No	92 (55%)	132 (41%)	
Yes	75 (45%)	188 (59%)	
Support a regulation to prohibit antibiotic prescription without laboratory confirmation of bacterial infection			0.4
No	56 (34%)	121 (38%)	
Yes	111 (66%)	199 (62%)	

^1^ n (%); ^2^ Pearson’s Chi-squared test.

**Table 8:** Association between experience, knowledge and attitude towards antibiotics.

Characteristic	5–10 Years, *n* ^1^ = 193	Less Than 5 Years, *n* = 219	More Than 10 Years, *n* = 75	*p*-Value ^2^
If antibiotics are taken as prescribed will this minimize the side effects				0.016
No	33 (17%)	19 (8.7%)	6 (8.0%)	
Yes	160 (83%)	200 (91%)	69 (92%)	
If antibiotics are taken without rational indication, will this increase the side effects				0.010
No	25 (13%)	18 (8.2%)	1 (1.3%)	
Yes	168 (87%)	201 (92%)	74 (99%)	
Do you think that previous use of antibiotic increases person risk of acquiring drug resistance infection				0.11
No	26 (13%)	16 (7.3%)	9 (12%)	
Yes	167 (87%)	203 (93%)	66 (88%)	
Feel under pressure if your patient expects antibiotic prescription				0.5
No	85 (44%)	109 (50%)	33 (44%)	
Yes	108 (56%)	110 (50%)	42 (56%)	
If your work load is high, are you more likely to prescribe antibiotics to relieve patient worry quickly				0.4
No	147 (76%)	166 (76%)	62 (83%)	
Yes	46 (24%)	53 (24%)	13 (17%)	
Think that if a prescription is issued, the consultation will be short				0.7
No	95 (49%)	117 (53%)	39 (52%)	
Yes	98 (51%)	102 (47%)	36 (48%)	
If you do not prescribe antibiotics, patients will feel their illnesses are not taken seriously				>0.9
No	70 (36%)	80 (37%)	26 (35%)	
Yes	123 (64%)	139 (63%)	49 (65%)	
Think that educating patient regarding antibiotic use will have an effect on their expectation in later consultation				0.9
No	27 (14%)	32 (15%)	9 (12%)	
Yes	166 (86%)	187 (85%)	66 (88%)	
Patient economic condition affects your selection of the prescribed antibiotics				0.6
No	44 (23%)	58 (26%)	21 (28%)	
Yes	149 (77%)	161 (74%)	54 (72%)	
Is there guidelines regarding antibiotic use in your institution				>0.9
No	23 (12%)	26 (12%)	10 (13%)	
Yes	170 (88%)	193 (88%)	65 (87%)	
If yes, do these guidelines affect your selection of the prescribed antibiotics				0.14
No	13 (7.7%)	27 (14%)	6 (9.4%)	
Yes	155 (92%)	165 (86%)	58 (91%)	
Unknown	25	27	11	
In my opinion, use of guidelines for antibiotics use is needed for proper patient care				0.5
No	15 (7.8%)	12 (5.5%)	3 (4.0%)	
Yes	178 (92%)	207 (95%)	72 (96%)	
The available guidelines for antibiotics use do not consider the individual variation of patients’ needs				0.2
No	98 (51%)	91 (42%)	35 (47%)	
Yes	95 (49%)	128 (58%)	40 (53%)	
Support a regulation to prohibit antibiotic prescription without laboratory confirmation of bacterial infection				0.8
No	71 (37%)	81 (37%)	25 (33%)	
Yes	122 (63%)	138 (63%)	50 (67%)	

^1^ n (%); ^2^ Pearson’s Chi-squared test; Fisher’s exact test.

**Table 9:** Multivariable Logistic Regression Analysis for Perceived Pressure to Prescribe Antibiotics.

Variable	Adjusted OR	95% CI	*p*-Value
Age (per year increase)	1.03	1.00–1.05	0.053
Male (vs Female)	1.05	0.72–1.53	0.795

## Data Availability

Data available on request.
